# A survey on multi-omics-based cancer diagnosis using machine learning with the potential application in gastrointestinal cancer

**DOI:** 10.3389/fmed.2022.1109365

**Published:** 2023-01-10

**Authors:** Suixue Wang, Shuling Wang, Zhengxia Wang

**Affiliations:** ^1^School of Information and Communication Engineering, Hainan University, Haikou, China; ^2^Department of Neurology, Affiliated Haikou Hospital of Xiangya School of Medicine, Central South University, Haikou, China; ^3^School of Computer Science and Technology, Hainan University, Haikou, China

**Keywords:** gastrointestinal cancer, multi-omics, machine learning, deep learning, integration

## Abstract

Gastrointestinal cancer is becoming increasingly common, which leads to over 3 million deaths every year. No typical symptoms appear in the early stage of gastrointestinal cancer, posing a significant challenge in the diagnosis and treatment of patients with gastrointestinal cancer. Many patients are in the middle and late stages of gastrointestinal cancer when they feel uncomfortable, unfortunately, most of them will die of gastrointestinal cancer. Recently, various artificial intelligence techniques like machine learning based on multi-omics have been presented for cancer diagnosis and treatment in the era of precision medicine. This paper provides a survey on multi-omics-based cancer diagnosis using machine learning with potential application in gastrointestinal cancer. Particularly, we make a comprehensive summary and analysis from the perspective of multi-omics datasets, task types, and multi-omics-based integration methods. Furthermore, this paper points out the remaining challenges of multi-omics-based cancer diagnosis using machine learning and discusses future topics.

## 1. Introduction

Cancer is one of the leading causes of death worldwide (1), usually with few symptoms in the early stage. However, once a patient is diagnosed with cancer, it is in the advanced stage of cancer. Cancer has a high morbidity and mortality rate worldwide and has become a common human disease, therefore, it poses a great threat to human beings. According to statistics (1), in 2020, there were about 19.3 million new cancer patients globally, and nearly 10.0 million patients died of cancer. Specifically, The number of new cases of breast cancer in the world reaches 2.3 million a year, becoming the most common cancer type globally, while 1.8 million cases of lung cancer deaths a year, rank first in the global cancer death population. Breast, lung, colorectal, stomach, liver, and prostate cancers are the most general types of cancer. Among them, all cancers in the digestive tract organs like colorectal cancer, gastric cancer, and liver cancer belong to gastrointestinal cancer, also becoming more and more common. In other words, the number of patients with these cancers is increasing every year. According to the institute for cancer research (1), some known risk factors such as smoking, unhealthy diet, being overweight and physical inactivity largely cause cancers, such as gastrointestinal cancer. It is extraordinarily difficult for doctors to diagnose and treat cancer patients, although surgery is one of the approaches to treatment for cancer patients, the recurrence rate is still high. Unfortunately, neither chemotherapy nor radiotherapy is ideal.

In order to improve the cancer treatment effect, as well as prolong the survival time for cancer patients, it is very essential to improve capabilities in precision medicine by using specific information about a patient's tumor to help make an accurate diagnosis, plan an effective treatment, find out how well treatment is working, or make a prognosis. In particular, accurate diagnosis can be used for the early diagnosis of cancer, many cancers can be cured if detected early and treated effectively (2). Even in the middle and advanced stages, being able to accurately diagnose cancer types or cancer molecular subtypes, also have a certain significance in enhancing treatment effects, improving the quality of life, and prolonging the life of patients.

**Figure 1:**
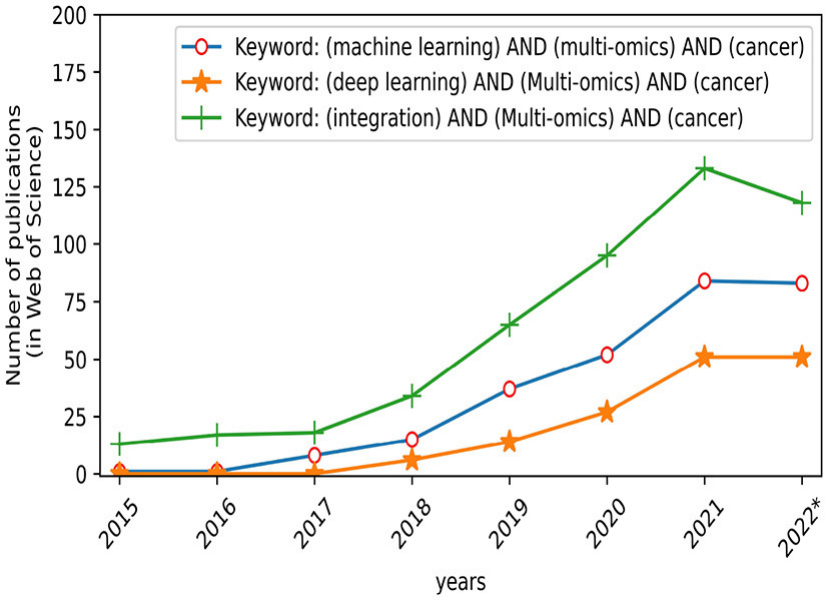
Number of publications published each year on different search keywords.

However, accurate diagnosis of cancer is a scientific problem in the field of biomedicine. Fortunately, over the past few years, with the development of artificial intelligence technology, especially machine learning (ML) and deep learning (DL) (3), smart medicine has developed rapidly (4). Smart medicine combines artificial intelligence technologies such as ML with medical theories and then applies them to pathological reports of cancer patients for converting cancer diagnosis into problems of classification, regression, or clustering. Especially in recent years, cancer diagnosis based on artificial intelligence has made great progress. As illustrated in Figure 1, the number of publications demonstrated that the multi-omics-based integrative methods using ML have become increasing interest in the area of cancer diagnosis over the last decade. For example, Stanford computer scientists have created an AI diagnostic algorithm that diagnosed skin cancer as well as a board-certified dermatologist (5). It is particularly emphasized that ML technology based on multi-omics is playing an important role in cancer diagnosis like survival analysis, drug sensitivity response, etc. (6, 7), and they have achieved corresponding curative effects on various cancers.

This paper provides a comprehensive review of multi-omics-based ML models or artificial intelligence technologies in the field of cancer diagnosis, and then we highlight its prospects and applications in gastrointestinal cancer. Finally, we point out the difficulties in the current multi-omics-based ML integration methods and discuss some future research directions.

**Figure 2:**
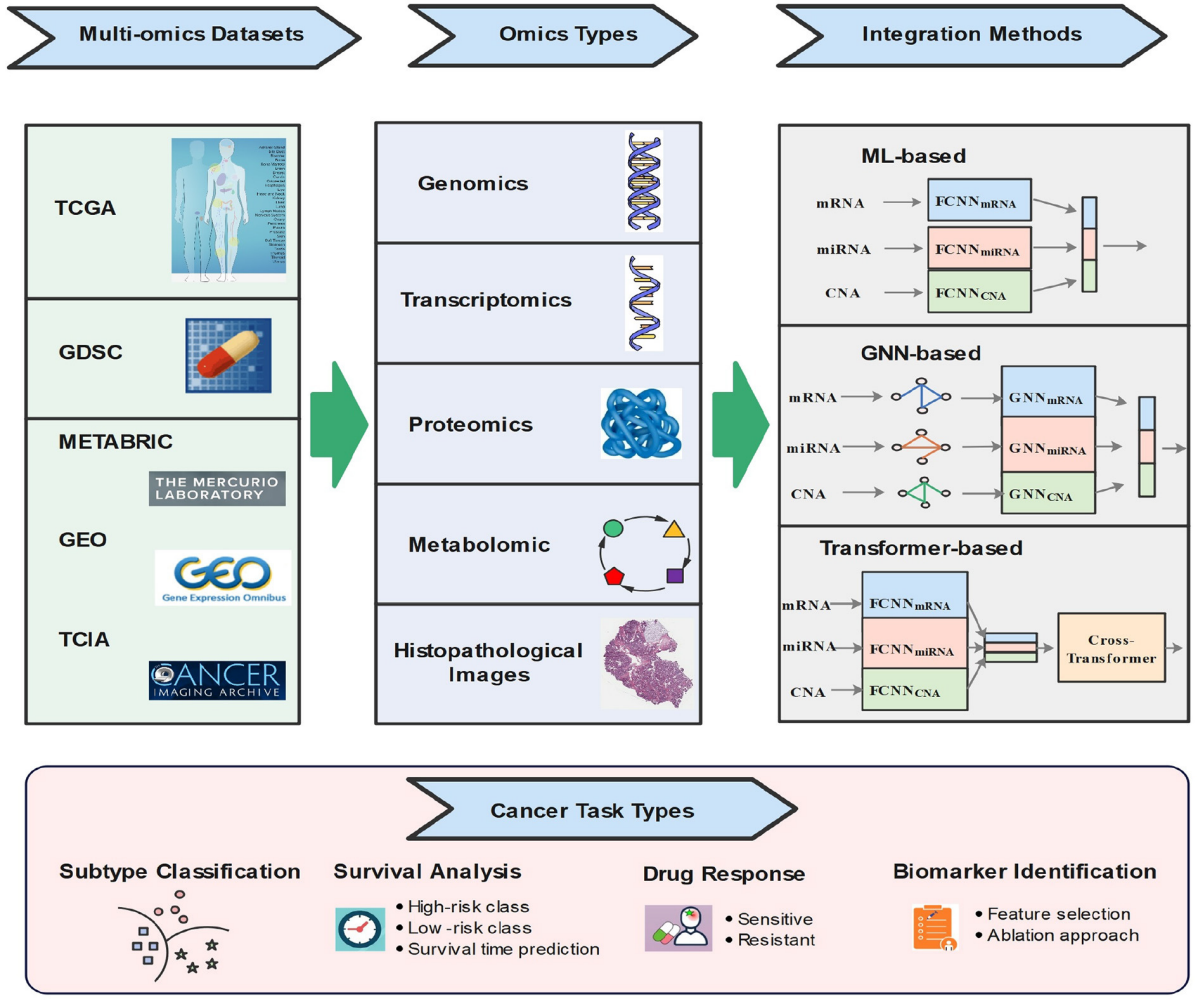
Illustration of multi-omics-based data integration using machine learning.

As shown in Figure 2, the rest of this paper is organized as follows. In Section 2, we detail the cancer task types based on multi-omics. We review some commonly used open-source omics databases in Section 3. Section 4 summarizes and discusses the state-of-the-art multi-omics-based ML integration methods for cancer diagnosis. Finally, Section 5 concludes the work and points out challenges and future directions.

## 2. Multi-omics-based cancer task types

Typical types of cancer tasks based on multi-omics data integration methods are cancer molecular subtype classification, survival analysis, drug response prediction, and biomarker discovery. In addition, some tasks are not well studied in the literature, such as metastasis prediction, recurrence prediction, etc., they will not be discussed in this review.

### 2.1. Molecular subtype classification and discovery of new subtypes

To customize the optimal treatment strategy for patients and achieve the purpose of precision medicine, it is of great significance to improve the accuracy of a cancer diagnosis. Specifically, cancer is generally further divided into multiple molecular subtypes, and different molecular subtypes adopt different treatment strategies to achieve the best therapeutic effect (8). For example, breast cancer is subdivided into four molecular subtypes: HER2-enriched, Luminal A, Luminal B, and Basal-like (9). Each subtype is associated with a unique panel of mutated genes. Therefore, the task of cancer subtype classification is to automatically identify defined subtypes based on the multi-omics measurement results of patients (10–13). In addition, with the growth of multi-omics data, there are still some potential cancer subtypes that need to be mined (14–16). Therefore, the identification of cancer subtypes is usually treated as a supervised classification problem, and the discovery of new subtypes is generally treated as a clustering problem.

### 2.2. Survival analysis

To improve the survival rate of cancer patients, a large number of researchers studied and analyzed the factors affecting their survival times by collecting the survival times of cancer patients and using machine learning methods to discover possible survival rules (17, 18). Cancer survival analysis can be defined as a binary classification or a risk regression problem (19, 20). In a binary classification task, patients are divided into short-term and long-term survival groups, or low-risk and high-risk survival groups, according to a predefined survival time threshold (e.g., 5 years) (21). In risk regression tasks, the Cox proportional hazards model and its extensions are often used to calculate the risk score for each patient.

### 2.3. Drug response prediction

IC50 is widely used to assess the sensitivity of drug response, and it is the concentration of drug required to reduce the number of viable cells by half after drug administration (22). Drug response prediction tasks are the same as survival analysis tasks. The binary classification tasks and regression tasks are often used in drug response research (19, 23–26). In regression problems, drug response directly predicts IC50 values, while in binary classification tasks, a predefined threshold based on the distribution of IC50 values is used to predict drug response as sensitive or resistant.

### 2.4. Biomarker discovery

In this review, the goal of biomarker discovery is to find genes associated with cancer prognosis by combining multi-omics data, which can advance the understanding of molecular mechanisms of cancer and offer new ideas for clinical diagnosis and treatment (27, 28). For example, in clinical practice in gastrointestinal cancer, CEA is the most commonly used marker. Furthermore, some biomarkers have been used for cervical cancer including miR-215-5p, miR-192-5p, KAT2B, PCNA, and CD86 (29). Biomarkers are widely identified by using the methods of feature selection and feature importance ranking in traditional ML (10, 30, 31). When analyzing the contribution of each feature in multiple omics sources, the feature will be set to 0 in turn, and then the performance of the classification or regression model will be calculated, and it will be compared to performance using all features (10).

## 3. Multi-omics datasets

It has become increasingly apparent that many novel omics data sequencing technologies have emerged since the Human Genome Project was proposed and implemented (32–34), and the cost of sequencing like high-throughput, is gradually decreasing. Therefore, we can quickly obtain high-dimensional multi-omics data and provide data sources for research in the fields of biomedicine and bioinformatics.

### 3.1. Multi-omics datasets

In this section, we introduce the multi-omics cancer datasets that are widely used in the literature. The multi-omics datasets are shown in Table 1.

**Table 1:** Frequently used multi-omics datasets.

**Dataset names**	**Data types**	**Supported task types**	**URL**
TCGA (35)	• Genomics • Transcriptomics • Epigenomics • Proteomics • Slide Image	• Subtypes classification • Biomarker discovery • Survival analysis • Drug response	https://portal.gdc.cancer.gov/
GDSC (36)	• Genomics • Drug response	• Drug response • Biomarker discovery	https://www.cancerrxgene.org/
METABRIC (37)	• Genomics	• Subtypes classification • Biomarker discovery	https://www.cbioportal.org/study/summary?id=brca-metabric
COSMIC Cell Lines (38)	• Genomics • Transcriptomics • Epigenomics • Drug response	• Subtypes classification • Biomarker discovery • Survival analysis • Drug response	https://cancer.sanger.ac.uk/cell-lines
CPTAC (39)	• Proteomics • Slide Image	• Subtypes classification • Biomarker discovery • Survival analysis	https://pdc.cancer.gov/pdc/
LinkedOmics (40)	• Genomics • Transcriptomics • Proteomics	• Subtypes classification • Biomarker discovery • Survival analysis	http://www.linkedomics.org/login.php
TCIA (41)	• Radiomics • Slide Image • Genomics	• Subtypes classification • Biomarker discovery • Survival analysis	https://www.cancerimagingarchive.net/

The Cancer Genome Atlas (TCGA) is a project jointly launched by the National Cancer Institute (NCI) and the National Human Genome Research Institute (NHGRI) in 2006 (35). It includes clinical information, histopathological images, and multiple omics data like genomics, transcriptomics, proteomics, and epigenomics. Especially, genomics and transcriptomics, are the most commonly used types of omics. For example, the data types of DNA methylation and copy number variation in genomics, as well as the data types of mRNA expression and miRNA expression in transcriptomics, appear most frequently in the literature on multi-omics-based ML integration methods. TCGA dataset currently includes a total of 33 types of cancer. In particular, all gastrointestinal cancer, including gastric cancer, colorectal cancer, liver cancer, etc., can be obtained in TCGA. TCGA is free and open, which greatly helps cancer researchers to improve the prevention, diagnosis, and treatment of cancer.

The Genomics of Drug Sensitivity in Cancer (GDSC) omics database was jointly developed by the Wellcome Trust Sanger Institute in the United Kingdom and the Massachusetts General Hospital Cancer Center in the United States (36). GDSC collects drug response data (IC50) of about 200 anticancer drugs in more than 1,000 human cancer cell lines. Variations in the cancer genome can affect the effectiveness of clinical treatment, and different targets have very different responses to drugs. Therefore, the GDSC data are really important for the discovery of potential tumor therapeutic targets, which have been widely used in anticancer drug screening.

In addition to the TCGA and GDSC datasets, other widely used databases also appear in the relevant literature, such as Molecular Taxonomy of Breast Cancer International Consortium (METABRIC), COSMIC Cell Lines, CPTAC, LinkedOmics, and the Cancer Imaging Archive (TCIA) (37–41).

### 3.2. Multi-omics challenges based on machine learning

Although multi-omics data can be used for cancer diagnosis using ML integration methods, there are still some problems with multi-omics data. We list some of the challenges that are quite general in the relevant literature (6, 42) as follows.

#### 3.2.1. Small sample size

The first challenge is that almost all existing omics datasets suffer from the problem of a small number of observations in a specific class, with most classes having < 100 observations. The features of omics usually have higher dimensionality, which is much larger than the number of observed samples, leading to the problem of the curse of dimensionality (43). In this case, it is crucial to use a reasonable evaluation method to estimate the classification error.

#### 3.2.2. Missing values

There are many missing values in clinical information and omics sequencing results in multi-omics datasets. Some studies have proposed that (17, 19) when a feature has more than 20% missing values in omics data, this feature will be discarded. At the same time, if our experimental content is to integrate and analyze multiple omics data, when patients lack any kind of omics data, the observation sample of this patient will also be discarded.

#### 3.2.3. Class imbalance

There is a problem of class distribution imbalance between different cancer types, as well as between different cancer molecular subtypes, respectively. To solve this problem, up-sampling and down-sampling techniques are usually employed (44).

## 4. Data integration methods for multi-omics using machine learning

In recent years, with the increase in computing power and the decline in the cost of high-throughput sequencing, and the success of ML technology in various fields, ML has been widely employed in the fields of biomedical and bioinformatics computing (45). In particular, a variety of novel data integration models have been introduced from ML. There are some reviews for summarizing the data integration methods based on multi-omics (6, 46), for example, the data are fused according to three strategies of early fusion, intermediate fusion, and late fusion (7). In addition, some reviews classify data integration methods according to concatenation-based, transformed-based, and model-based approaches (42). In this section, we classify the newly proposed data integration models according to three types of groups: traditional ML-based, transformer-based, and graph neural network based.

### 4.1. Conventional machine learning technologies

Here we will briefly introduce three subgroups of models applied in data integration: traditional ML models, classical deep learning models, and auto-encoder models.

Logistic regression (LR), support vector machine (SVM), random forest (RF) and Xgboost are widely used traditional ML models (30, 31, 47), before feeding the data to them, it generally needs to reduce the dimensionality of high-dimensional features of multiple omics data based on feature extraction methods such as nearest component analysis (NCA) (19, 23) and principal component analysis (PCA) (21), and then concatenate the dimensionality-reduced features and finally feed the concatenated features to the model.

In contrast, it is not necessary for classical deep learning models like fully connected neural networks (FCNNs) and convolution neural networks (CNNs) to reduce the dimensionality of omics features to very low dimensions, due the models can automatically learn useful information from high-dimensional space (11, 48–51).

Auto-encoder is an unsupervised neural network model where the network can be replaced by FCNN, CNN, or other DNNs (16). Auto-encoder compresses the data to a lower dimension, which is called encoding, and then reconstructs the original input data back, which is called decoding (11). Intuitively, auto-encoder can be used for dimensionality reduction which is similar to PCA, but its performance is stronger than PCA due to the neural network model can extract more effective new features (52). In addition to dimensionality reduction, new features learned by the auto-encoder can be fed into the supervised learning model for the tasks of classification or regression.

### 4.2. Graphic neural network technologies

In recent years, Graph Neural Networks (GNNs) have shown strong capabilities in handling non-Euclidean graph-structured data by naturally combining network topology structure and the information of node and link, GNNs have been employed to integrate multi-omics data since the last 2 years. Wang et al. (10) proposed MOGONET, for each omics data type, a weighted sample similarity network was constructed according to omics characteristics as the input of GNN and used for the identification of biomarkers. In Xing et al. (53), MLE-GAT is presented to explore the correlation information between genes contained in omics data. It assumes that genes usually interact rather than acting alone, so the weighted correlation network analysis (WGCNA) is firstly used to convert each patient's omics data into a co-expression map as the input of GAT, and then GAT outputs each node feature as a weighted combination of its neighbors and the current node.

### 4.3. Transformer technologies

The transformer model is widely used in different fields such as natural language processing and computer vision, it is becoming one of the most frequently used deep learning models (54–56). The success of the transformer architecture depends on the multi-head attention mechanism that calculates the attention between different positions in the input sequence multiple times. The Transformer is applied to multi-omics-based integration techniques since 2021, which is a relatively new data integration method. For example, in these two papers (57, 58), the transformers are used to calculate the cross-attention between multi-omics features and histopathological image features.

## 5. Conclusion

In this paper, we review the multi-omics-based integrative approaches using ML with potential applications in gastrointestinal cancer. Firstly, several cancer task types are elaborated on and discussed. Then we describe widely used cancer multi-omics datasets, and the challenges encountered in their use for integration based on ML. Finally, we analyze currently the state-of-the-art multi-omics-based data integration approaches in detail and divide them into three groups such as conventional ML technologies, graphic neural network technologies, and transformer technologies.

Although ML has performed excellently in the application of multi-omics data integration, there are still some challenges that require us to consider and explore deeply. Specifically, the existing methods for a missing value of multi-omics data are almost all treated as discards, rather than trying to fill them in. Therefore, to efficiently utilize the existing precious multi-omics data, it is necessary to further explore the method of filling in missing values in multi-omics. Additionally, since biomedical data are very precious and difficult to obtain, the patients who contain multiple omics sources and histopathology images simultaneously are particularly scarce. Hence, in the future, a pre-trained visual representation model may be transferred to histopathology images on a limited number of samples, which can be potentially solved by few-shot learning strategies. More importantly, more effective approaches for integrating multi-omics and histopathology images need further investigation for gastrointestinal cancer diagnosis and treatment, as a promising future research direction.

## Author contributions

ShW contributed to the conception of the study and the verification of analytical methods. SuW performed the statistical analysis and wrote the manuscript. ZW revised the manuscript and approved the version to be published. All authors contributed to the article and approved the submitted version.
